# Parental Regret Following Decision to Revise Circumcision

**DOI:** 10.3389/fped.2022.855893

**Published:** 2022-03-09

**Authors:** Noam Bar-Yaakov, Roy Mano, Margaret Ekstein, Ziv Savin, Snir Dekalo, Jacob Ben-Chaim, Yuval Bar-Yosef

**Affiliations:** ^1^Pediatric Urology Department, Dana-Dwek Children's Hospital, Tel-Aviv Medical Center, Tel-Aviv, Israel; ^2^Sackler School of Medicine, Tel-Aviv University, Tel-Aviv, Israel

**Keywords:** penis, circumcision, decision making, surgery, outcome

## Abstract

**Purpose:**

Revision surgery for the removal of excess foreskin after circumcision is a common procedure. The decision regret scale (DRS) is a validated questionnaire which assesses regret after medical decision making. The aim was to evaluate parental regret by means of the DRS and querying about factors associated with regret about deciding to revise their child's circumcision.

**Patients and Methods:**

Included were all pediatric patients who underwent revision of neonatal circumcision in a single center between 2010 and 2016. Excluded were children who underwent revision for reasons other than excess foreskin, those who underwent additional surgical procedures during the same anesthetic session, and those who had undergone previous penile surgery other than circumcision. Response to the DRS questionnaire was by a telephone call with the patient's parent. Regret was classified as none (a score of 0), mild (1–25), or moderate-to-strong (26–100). Surgical and baseline demographic data were obtained from the departmental database and compared between the no regret and regret groups.

**Results:**

Of the 115 revisions of circumcisions performed during the study period, 52 fulfilled the inclusion criteria, and the parents of 40 (77%) completed the DRS questionnaire. Regret was reported by 11/40 [28%: nine as mild (23%) and two as moderate-to-strong (5%)]. The average age of the child in the regret group was 17 months compared to 18 months in the no regret group (*p* = 0.27). The median weight percentile was 43% in both groups. Surgical variables, including anesthesia type (caudal vs. no block, *p* = 0.65), suture type (polyglactin vs. poliglecaprone, *p* = 0.29), operation time (28 vs. 25 min, *p* = 0.59), and anesthesia time (55 vs. 54 min, *p* = 0.57) were not significantly different between the groups.

**Conclusions:**

Regret for deciding upon revision surgery for removal of excess foreskin post-circumcision was reported by 27.5% of parents of children who underwent revision. No clinical, surgical, or demographic characteristics predicted parental decisional regret.

## Introduction

Neonatal male circumcision is a common procedure worldwide and nearly universal in our country. It is often performed by a traditional circumciser or, alternatively, by a medical professional. The complications associated with circumcision are mostly mild, and they include pain, edema, minor bleeding, and excess foreskin (1), while more serious complications, such as glans injury, urethral injury, and massive bleeding are rare (2, 3). Surgical interventions for the more serious complications as well for minor complications, such as penile skin bridges and inclusion cysts, are indicated for functional reasons. Revision surgery for excess and redundant foreskin is undertaken mainly for cosmetic issues. While the reduction of excess foreskin is a safe and simple procedure (4, 5), it is nonetheless an elective surgical intervention carried out under general anesthesia with risk of associated complications, chosen by caregivers for their children.

Any pediatric surgery can be associated with parental distress and fear of surgical and anesthetic complications. In addition, parents may not be satisfied with the course of an operative procedure, beginning with the fasting period before surgery, the time spent in the operating room, the recovery period in the hospital and later at home, and finally with the outcome of surgery. The decision may be more difficult when the indication for surgery is entirely cosmetic and not functional.

The decision regret scale (DRS) is a validated questionnaire which assesses regret after a medical intervention. It has been studied in a variety of surgical procedures and is associated with patient satisfaction as well as quality of life (6). The DRS has been used for assessing parental regret after pediatric surgery in urology as well as in other fields (7). Several studies have shown 50–90% parental regret after distal hypospadias repair (8–10), but, to the best of our knowledge, parental regret following revision of circumcision has not been investigated. The primary objective of this study was to evaluate decisional regret on the part of the parents who consented to surgery for the removal of excess foreskin following circumcision and who responded to the DRS questionnaire. The secondary objective was to identify factors associated with decisional regret.

## Patients and Methods

With the approval of the institutional review board, we identified all male children who underwent repair of neonatal circumcision in our tertiary care center between 2010 and 2016. Excluded were children who underwent revision of circumcision for reasons other than excess foreskin, those who underwent additional surgical procedures in the same anesthetic session, and those who had undergone previous penile surgery other than circumcision.

The procedures were performed with the patient under general anesthesia and without the use of perioperative antibiotics. A distal circumferential incision under the corona and a second one proximally to it were marked and performed. The excess skin sleeve was then incised dorsally and removed by means of cautery, along with the underlying subcutaneous tissue. The skin edges were approximated with absorbable sutures (6/0 polyglactin or 5–6/0 poliglecaprone according to the surgeon's preference). The patients were discharged on the same day, and the routine postoperative evaluation included an outpatient clinic visit 4 weeks after the surgery.

The medical charts of children who underwent repair of circumcision and fulfilled the inclusion criteria were retrieved from the departmental database and reviewed, and the following data were collected: age at procedure, weight percentile, operation time, operating room time, anesthesia type, suture type, length of stay in hospital, and complications. The parents of the enrolled children were contacted telephonically between July 2017 and August 2017, and the consenting parents responded to a DRS questionnaire. Decisional regret was classified as none (a score of 0), mild (1–25), and moderate-to-strong (26–100). If both parents of the same child answered the DRS, their DRS scores were averaged and recorded. The time between the operation and the evaluation by the DRS was recorded as well.

Data on the clinical characteristics of the study cohort were reported by means of descriptive statistics. Continuous variables were reported by the median and interquartile range (IQR), and categorical variables were reported by number and percent. Findings were compared between the children of parents with and without decisional regret by means of the Wilcoxon rank sum test for continuous variables and Fisher's exact test for categorical variables. All statistical analyses were two-sided, and significance was defined as *p* < 0.05. All analyses were conducted with R Statistical Software (version 4.1.1; R Foundation for Statistical Computing, Vienna, Austria).

## Results

A total of 115 male children underwent revision of their circumcision during the study period, and 52 of them fulfilled the inclusion criteria and comprised the study cohort. DRS scores were obtained for 40/52 patients (a 77% response rate), with reports from 53 parents. Patients and procedure characteristics for the cohort are reported in Table 1. The median age at surgery was 18 (IQR 12.75, 36) months, the median weight percentile was 43 (IQR 19, 81), the anesthesia type was caudal combined with general in 33 patients (83%), and the median operative time was 27 min (IQR 20, 32). All of the patients were discharged on the same day of the procedure and there were no reports of postoperative complications or 30-day readmissions.

**Table 1:** Patients' clinical characteristics.

**Variable**		**No. (*n* = 40)**
Age, months (median [IQR])		18 [12.75, 36]
Weight percentile (median [IQR])		43 [16, 81]
Suture type (%)	Polyglactin	21 (52.5)
	Poliglecaprone	19 (47.5)
Anesthesia type (%)	General	7 (17.5)
	General + caudal	33 (82.5)
OR time, min (median [IQR])		54.5 [46.75, 65.25]
Operation time, min (median [IQR])		27 [20, 32]
Length of stay (days)		1
Time from surgery to evaluation, months (median [IQR])		25 [16.75, 37.25]

*IQR, interquartile range; OR, operating room*.

Parental decisional regret according to the DRS was reported in 11 cases (28%), nine of which were mild (23%) and 2 were moderate-to-strong (5%). The average DRS for this group of parents was 7.98. The time between surgery and the evaluation of DRS did not differ significantly between the patients whose parents did not report decisional regret and those who did (median 24 vs. 26 months, respectively). Table 2 summarizes the clinical characteristics of the regret vs. no regret group: none of the investigated variables predicted decisional regret, including age at surgery, weight percentile, suture material, and anesthesia type. Operating room time and operation duration also did not differ significantly between the two groups.

**Table 2:** Clinical characteristics of the study cohort stratified by parental decisional regret (*n* = 40).

**Variable**		**Decisional regret**		***p*** -value^*^
		**No (*n* = 29)**	**Yes (*n* = 11)**	
Age, years (median [IQR])		18 [14, 42]	17 [11.5, 24]	0.268
Weight percentile (median [IQR])		43 [16, 81]	43 [29, 78.5]	0.524
Suture type (%)	Polyglactin	17 (58.6)	4 (36.4)	0.293
	Poliglecaprone	12 (41.4)	7 (63.6)	
Anesthesia type (%)	General	6 (20.7)	1 (9.1)	0.65
	General + caudal	23 (79.3)	10 (90.9)	
OR time min (median [IQR])		55 [47, 65]	54 [48.50, 70.5]	0.565
Operation time min (median [IQR])		28 [20, 32]	25 [19.50, 31.5]	0.585
Time from surgery to evaluation months (median [IQR])		24 [17, 33]	26 [20, 38.5]	0.396

* *Categorical variables were compared with Fisher's exact test and continuous variables with the Wilcoxon rank sum test. IQR, interquartile range; OR, operating room*.

## Discussion

There is little evidence about the factors that influence parental choices of treatment for their children and what promotes appropriate decisions that lead to good medical outcomes and lack of decisional regret (11). Outcome research in all fields of medicine, pediatric urology included, had been mainly based upon endpoints, such as survival, complications, and reoperation and readmission rates. Patient satisfaction was later included as an outcome measure. There is a paucity of information on parental decision making processes and on the satisfaction or regret associated with these decisions in the field of pediatric urology (12).

The publications on parental regret that are available have focused mainly upon hypospadias surgery, and some have looked at these issues following surgery for fecal incontinence as well as feminizing genitoplasty in congenital adrenal hyperplasia (13, 14). Ghidini et al. used the DRS to investigate parental regret following the decision to repair distal hypospadias. Data were available for 172 of 372 families. Of the 323 returned questionnaires, 128 (39.6%) reported moderate-to-strong regret, 169 (52.3%) reported mild regret, and only 26 (8.1%) reported no regret. Factors which predicted moderate-to-strong regret included parent education level, patient not being the firstborn, family history of hypospadias, initial desire to avoid surgery, younger age at follow-up, presence of lower urinary tract symptoms, and lower pediatric penile perception score. Those authors observed that moderate-to-strong regret was unrelated to surgical variables, the development of complications, or the duration of follow-up (9).

van Engelen et al. reported mild regret in 39.2% of 97 participating parents, and moderate-to-strong regret in 11.3%, for a total regret rate of 50.5% after distal hypospadias repair. Psychosocial behavior problems of the child and decisional conflict significantly predicted decisional regret, while demographic and medical variables did not (10). Lorenzo et al. similarly evaluated parental regret following distal hypospadias repair: 48 of 116 participants (41.4%) reported mild regret and 10 (8.6%) reported moderate-to-strong regret for a total of 50% of parents reporting some level of regret. In contrast to the other publications, complications were the strongest predictor of moderate-to-strong regret, and that parameter also correlated with parental desire to avoid circumcision and the level of their decisional conflict (8).

The lower complication rate of revision of circumcision compared to distal hypospadias repair is a highly likely reason for the lower rate of regret in our survey. Importantly, there were no complications that required additional surgical intervention. The only complication we encountered was skin adhesion that was freed uneventfully in a clinic visit. This low complication rate is in line with previous studies of circumcision revision. Redman et al. reported 46 cases of circumcision revision with no complications (5) and Brisson et al. reported one (1.8%) emergency surgery after circumcision revision due to surgical site bleeding (4).

Parents are concerned about the appearance of a penis with excess skin which may resemble an uncircumcised penis. Often the concern is with the reactions of future sexual partners or of peers, the latter especially relevant in common showers during compulsory military service. This concern by parents is supported by past research. Alexander et al. met similar concerns voiced by parents presenting with their sons for circumcision revision: they were apprehensive that their child may be teased later in life in school locker rooms because of the appearance of his penis. Using questionnaires presented to 290 male university undergraduate students, they found that almost 50% of the boys had observed someone else being teased about penile appearance in school locker rooms, and 10% reported being teased themselves (16).

There are several limitations to be considered when interpreting our results. The data were collected retrospectively *via* a telephone call. The documented responses were provided by only one parent for most of the children, thus not reflecting a possible decisional conflict between the parents (15), or different regret following the procedure. The sample size of our study is small, thus possibly affecting the ability to identify a wider range of factors which could predict parental decisional regret. Most significantly, the results are from a single center in a country where circumcision is almost universal for cultural and religious reasons, it is possible that our results are not generalizable to countries where circumcision is less commonplace.

## Conclusions

Decisional regret as reflected by the DRS questionnaire was reported by 27.5% of parents of children who underwent revision of neonatal circumcision for excess foreskin. No clinical, surgical, or demographic characteristics predicted parental decisional regret.

## Data Availability Statement

The raw data supporting the conclusions of this article will be made available by the authors, without undue reservation.

## Ethics Statement

The studies involving human participants were reviewed and approved by Tel-Aviv Medical Center Institutional Review Board. Written informed consent to participate in this study was provided by the participants' legal guardian/next of kin.

## Author Contributions

NB-Y, JB-C, and YB-Y contributed to conception and design of the study. NB-Y, ZS, ME, and SD contributed to data collection and organized the database. RM performed the statistical analysis. NB-Y wrote the first draft of the manuscript. RM and YB-Y wrote sections of the manuscript. All authors contributed to manuscript revision, read, and approved the submitted version.

## Conflict of Interest

The authors declare that the research was conducted in the absence of any commercial or financial relationships that could be construed as a potential conflict of interest.

## Publisher's Note

All claims expressed in this article are solely those of the authors and do not necessarily represent those of their affiliated organizations, or those of the publisher, the editors and the reviewers. Any product that may be evaluated in this article, or claim that may be made by its manufacturer, is not guaranteed or endorsed by the publisher.
